# Aggregation Condition–Structure Relationship of Mouse Prion Protein Fibrils

**DOI:** 10.3390/ijms22179635

**Published:** 2021-09-06

**Authors:** Jēkabs Fridmanis, Zigmantas Toleikis, Tomas Sneideris, Mantas Ziaunys, Raitis Bobrovs, Vytautas Smirnovas, Kristaps Jaudzems

**Affiliations:** 1 Department of Physical Organic Chemistry, Latvian Institute of Organic Synthesis, Aizkraukles 21, LV-1006 Riga, Latvia; fridmanis.jekabs@osi.lv (J.F.); zigmantas.toleikis@gmc.vu.lt (Z.T.); raitis.bobrovs@osi.lv (R.B.); 2 Institute of Biotechnology, Life Sciences Center, Vilnius University, LT-10257 Vilnius, Lithuania; sneideris.t@gmail.com (T.S.); mantas.ziaunys@gmc.vu.lt (M.Z.); vytautas.smirnovas@bti.vu.lt (V.S.)

**Keywords:** amyloid, prion protein, fibril structure, aggregation conditions

## Abstract

Prion diseases are associated with conformational conversion of cellular prion protein into a misfolded pathogenic form, which resembles many properties of amyloid fibrils. The same prion protein sequence can misfold into different conformations, which are responsible for variations in prion disease phenotypes (prion strains). In this work, we use atomic force microscopy, FTIR spectroscopy and magic-angle spinning NMR to devise structural models of mouse prion protein fibrils prepared in three different denaturing conditions. We find that the fibril core region as well as the structure of its N- and C-terminal parts is almost identical between the three fibrils. In contrast, the central part differs in length of β-strands and the arrangement of charged residues. We propose that the denaturant ionic strength plays a major role in determining the structure of fibrils obtained in a particular condition by stabilizing fibril core interior-facing glutamic acid residues.

## 1. Introduction

Protein aggregation into insoluble, beta-sheet rich fibrillar aggregates [1] is linked with the onset and progression of several amyloidosis [2], including neurodegenerative disorders, such as Alzheimer‘s, Parkinson‘s or prion diseases [3,4]. Years of research into this type of protein fibrillization has resulted in a general understanding of the steps and mechanisms involved in the transition from native-state protein molecules to beta-sheet rich and highly-structured aggregates [5,6,7]. However, the overall process is still not fully understood. A peculiar aspect of amyloid formation is the ability of one type of protein/peptide to form multiple, structurally distinct fibrils [8] and this property has been observed for several amyloidogenic proteins, ranging from the Alzheimer‘s disease-related amyloid beta peptide [9,10] to model amyloid proteins, such as insulin [11] or lysozyme [12]. Lack of insight into this, seemingly generic, property of protein fibrillization may be one of the reasons why there is still no complete understanding of amyloid aggregation and, in turn, very few effective treatments for their respective diseases [13,14,15].

A prime example of such polymorphic nature is the prion protein [16], a glycoprotein, whose aggregation into amyloid fibrils is associated with transmissible spongiform encephalopathies, such as Creutzfeldt-Jakob disease, Gerstmann–Straussler–Scheinker syndrome or fatal familial insomnia [17]. These structural variations can define multiple factors of transmission and propagation, including susceptibility [18], incubation period [19] and disease phenotype [20]. The resulting fibrils can also possess specific morphological or secondary structure motifs, such as length [21], diameter [22], number of intertwined protofilaments [23], periodicity patterns [24], proteinase K resistant cores [25] or beta-sheet content [26]. Despite the already intricate nature of prions observed in vivo, matters become even more complicated in vitro.

It has been observed in a number of studies that the environment, under which protein aggregation occurs, has a profound effect on the resulting structure and morphology of amyloid fibrils. Various factors, including pH [27], agitation [10], protein concentration [28], ionic strength [29] or denaturant concentration [30,31] can lead to the formation of a different type of fibril. This has been the case for many widely tested amyloidogenic proteins [9,32,33]. Taking into consideration that in vitro studies are not all carried out under identical experimental conditions, the resulting aggregates may also be completely different. In such a situation, results obtained from one study cannot be accurately compared to another, as the generated fibrils may have specific rates of propagation, stabilities against fragmentation or denaturation and self-association properties.

One means of combating this problem is to determine the condition–structure relationship between the environment used to generate prion fibrils and their type. However, this is complicated by the fact that such aggregates are insoluble and non-crystalline, thus greatly limiting conventional protein structure assays. Transmission electron [34] or atomic force microscopies [35] only provide morphological details about the aggregate, while Fourier-transform [36] or Raman spectroscopies [37] give an average overview of their secondary structure. Magic-angle spinning solid-state nuclear magnetic resonance (MAS NMR) of full-length prion protein has allowed identification of the structurally ordered fibril core, but spectral resolution was not sufficient for residue- or atomic-level studies [38]. Until recently, the only experimentally determined prion protein fibril structure was derived from electron paramagnetic resonance (EPR) spectroscopy data [39]. Last year, two high-resolution cryo-electron microscopy (cryo-EM) structures of wild-type (wt) and E196K mutant human prion protein (HuPrP) fibrils have been published [40,41]. Although both fibrils were obtained in identical conditions, the fibril structure is markedly different, highlighting the impact of a point mutation (i.e., charge reversal) on the prion protein aggregation pathway. However, high resolution structures of prion fibrils produced in different environments, which are essential for deciphering the condition–structure relationship, are still lacking.

In this work, we investigate mouse prion protein folding domain (MoPrP(89-230)), which corresponds to proteinase K-resistant part of pathogenic PrP, and is often used for in vitro studies [42]. We prepare fibrils in three different denaturing conditions and study them using Fourier-transform infrared spectroscopy (FTIR), atomic force microscopy (AFM) and MAS NMR. Based on the NMR chemical shift assignments we compare locations of secondary structure elements. Finally, we analyze our obtained data in conjunction with the three known structures of human prion protein fibrils to determine the structural implications of aggregation conditions.

## 2. Results and Discussion

Recombinant MoPrP(89-230) was expressed in *Escherichia coli* and purified as described in the Materials and Methods section. Since spontaneous aggregation of recombinant prion protein is too slow under ambient conditions, the addition of denaturing agents is required. Commonly used prion protein aggregation conditions include either 2 or 4 M guanidinium hydrochloride (GdnHCl) in phosphate buffer solution [43,44,45] as the sole denaturant or a mix of 3 M urea and 1 M GdnHCl in PBS [46,47,48]. Besides the composition of denaturing agents, the aforementioned aggregation reaction solutions can have different pH values, as well as agitation conditions. To study the impact of the aggregation conditions on the structure of mouse prion fibrils, we prepared aggregates in 2 M GdnHCl with 50 mM sodium phosphate, pH 6.0 (G2 condition), 4 M GdnHCl with 50 mM sodium phosphate, pH 6.0 (G4 condition) and a mixture of 3 M urea and 1 M GdnHCl with 1× PBS, pH 7.4 (U3G1 condition). These conditions were chosen because they have been used in multiple previous studies [43,44,45,46,47,48] and are well documented in terms of aggregation kinetics and fibril morphology. Reseeding was used to minimize fibril sample heterogeneity and select for the most stable structure under each of the conditions. The obtained fibrils were characterized by FTIR spectroscopy, AFM, and their structure was studied in detail by MAS NMR spectroscopy.

### 2.1. Fibril Secondary Structure and Morphology Assessed by FTIR and AFM

**Figure 1:**
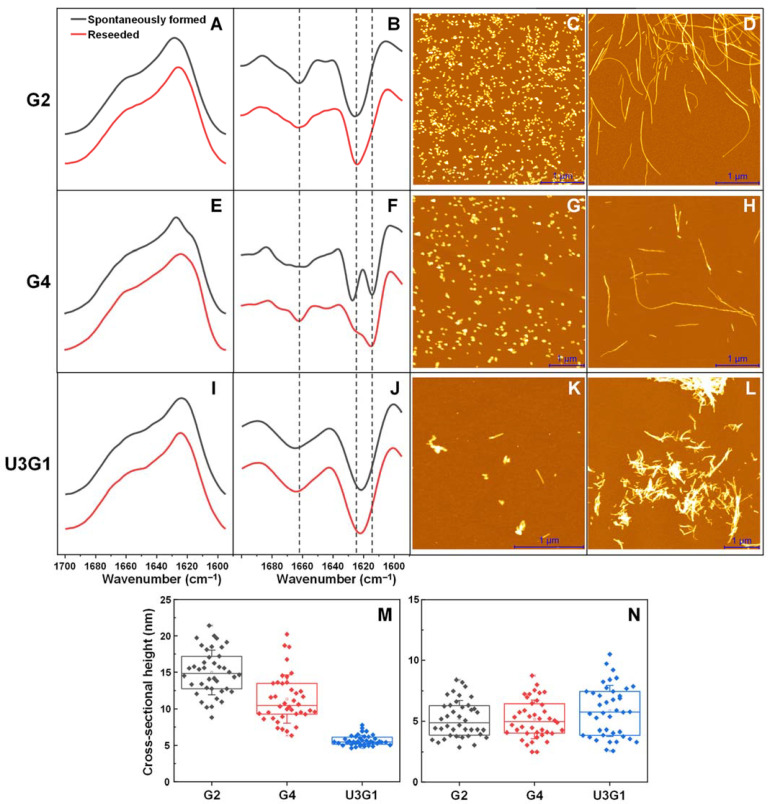
FTIR spectra and AFM images of spontaneously formed and reseeded MoPrP(89-230) fibrils. FTIR spectra and their second derivatives of G2 (**A**,**B**), G4 (**E**,**F**) and U3G1 (**I**,**J**) condition fibrils. AFM images of spontaneously formed (**C**,**G**,**K**) and reseeded (**D**,**H**,**L**) G2, G4 and U3G1 condition fibrils respectively. Cross-sectional heights of spontaneously formed (**M**) and reseeded fibrils (**N**).

The spontaneously formed and reseeded prion protein fibrils were examined using FTIR spectroscopy and AFM in order to determine structural and morphological differences between aggregates formed under all three conditions and study effects of reseeding. Upon reseeding, the G2 condition fibrils retain a similar FTIR spectrum (Figure 1A,B) with a main maximum at 1624 cm^−1^ (main minimum in the second derivative spectrum), indicating one dominant type of hydrogen bonding in the beta-sheet structure [49]. The morphology changes from small, clumped aggregates (Figure 1C), with an average cross-sectional height of 15 nm (Figure 1M), to relatively long fibrils (Figure 1D) with a height of 5 nm (Figure 1N). Conversely, the G4 condition aggregates did experience a change in their secondary structure upon reseeding (Figure 1E,F). The spontaneously formed aggregates had a FTIR spectrum maximum at 1628 cm^−1^ and two clearly expressed minima (1628 and 1614 cm^−1^) in the second derivative spectrum, indicating the presence of two types of hydrogen bonding. When the aggregates were reseeded, the main maximum shifted to 1614 cm^−1^ and the second derivative main minimum at 1614 cm^−1^ only had a shoulder at 1628 cm^−1^. Such a change is associated with the decreased proportion of weaker hydrogen bonds in the beta-sheet structure. Reseeding also caused the fibrils to become highly elongated (Figure 1G,H) and the cross-sectional height experienced a similar, yet less significant change to 5 nm (Figure 1M,N).

Similarly to G2, the U3G1 condition fibril FTIR spectra did not have any notable changes upon reseeding (Figure 1I,J), with a main maximum at 1621 cm^−1^, which means that the beta-sheet structure contains one dominant type of hydrogen bonding, which is slightly stronger than in the case of G2 fibers. The spontaneously formed aggregates had a significantly smaller cross-sectional height (5 nm, Figure 1M) and, oppositely to both G2 and G4, reseeding did not cause substantial changes to either fibril length or height (Figure 1K–N). The considerably shorter length of U3G1 aggregates, as compared to G2 and G4, may indicate a lower structural stability (higher rate of fragmentation) or a higher incidence of surface-mediated nucleation events. For all three conditions, there is one minimum in the second derivative FTIR spectrum, associated with turn/loop motifs (1660–1680 cm^−1^) [49], however, it is shifted towards a lower wavenumber position in the case of U3G1 fibrils, hinting at another possible structural difference between aggregates formed using only one type of denaturant. Altogether, these data corroborate previous findings that the morphology and structure of MoPrP(89-230) fibrils is dependent on the aggregation conditions. The reseeding altered morphology of G2 and G4 aggregates but did not have a profound effect on their secondary structure, except for a population shift towards the more stable of two conformations formed under the G4 condition.

### 2.2. MAS NMR Fingerprints

Initial MAS NMR spectra of uniformly (U)-^13^C,^15^N-labeled MoPrP(89-230) fibrils spontaneously aggregated in U3G1 condition (without reseeding) showed poor resolution (Supplementary Figure S3) similar to that seen for Syrian hamster PrP(23-231) fibrils [38]. To increase spectral homogeneity, a six-generation reseeding procedure was employed to prepare the fibrils for NMR. This led to spectra with improved quality, although still displaying a significant overlap of peaks, particularly in the threonine and valine region. To reduce the spectral crowding, further sample preparations were done by using amino acid-selective unlabeling of threonines and arginines, as described in the Materials and Methods section (see also Supplementary Figures S1 and S2).

**Figure 2:**
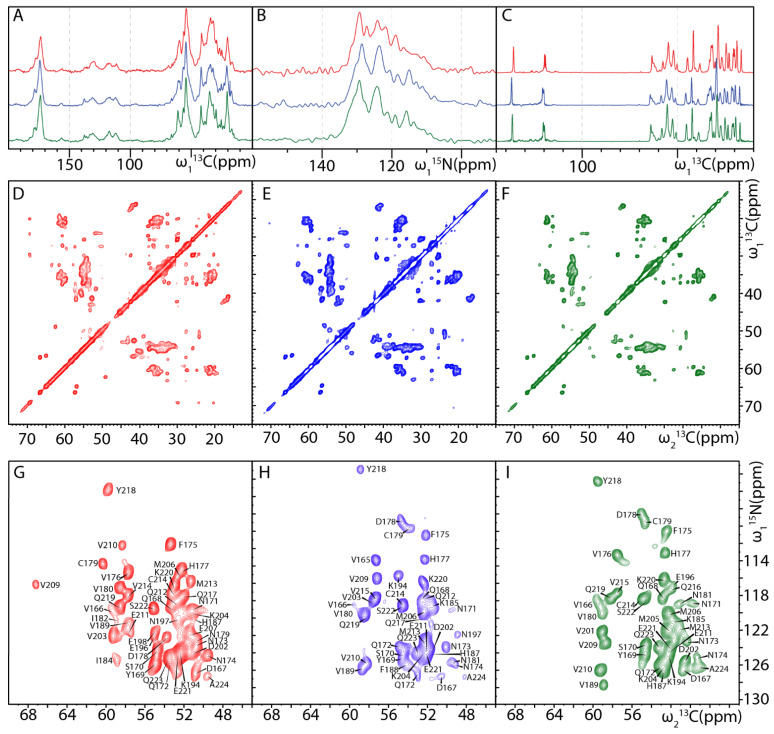
MAS NMR spectra of U-^13^C,^15^N-labeled Thr-, Arg-selectively unlabeled MoPrP(89-230) fibrils. Spectra of fibrils aggregated in U3G1 condition are colored red, in G2 condition—blue and in G4 condition—green. 1D ^13^C CP (**A**), ^15^N CP (**B**), ^13^C INEPT spectra (**C**), 2D DARR spectra (**D**–**F**) and assigned 2D NCA spectra (**G**–**I**) were recorded at 800 MHz and 273 K with 12.5 kHz MAS. Due to sequence discrepancy between mouse and human prions, in this figure and further, human prion protein residue numbering is adapted.

Figure 2 shows a comparison of 1D ^13^C CP, ^15^N CP, ^13^C INEPT and 2D DARR and NCA spectra of U-^13^C,^15^N-labeled, Thr-, Arg-unlabeled MoPrP(89-230) fibrils produced at the three above-mentioned aggregation conditions. Their side-by-side comparison suggests a higher similarity between fibrils produced in presence of GdnHCl only (G2 and G4 conditions), whereas the fingerprints of fibrils obtained in U3G1 condition indicate a significantly altered fibril structure. The most striking difference is a low-field-shifted ^13^C_α_ resonance that was assigned to Val209. These results suggest that the denaturing agent type is more important than concentration in determining the final fibril structure. ^13^C INEPT spectra show many peaks from mobile polypeptide regions, indicating that the fibrillar core is constituted of only a portion of the amino acid sequence. However, almost no differences between the ^13^C INEPT spectra of the three samples were observed, suggesting that the fibrillar cores are confined to approximately the same protein region.

### 2.3. Locations of Secondary Structure Elements

**Figure 3:**
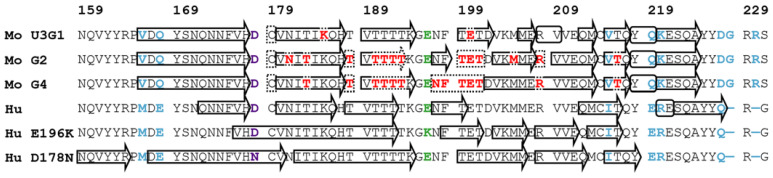
Comparison of secondary structures between mouse (Mo) and human (Hu) prion protein fibrils. The secondary structures of MoPrP(89-230) U3G1, G2 and G4 condition fibrils were derived from secondary chemical shift analysis (see Supplementary Figure S5). Beta sheets are indicated with an arrow and residues with helical torsion angles are encircled with a rounded rectangle. Residues for which the secondary structure could not be determined are indicated with dots. Residues with missing assignments are colored red, sequence discrepancies between HuPrP and MoPrP sequences are colored blue, E196K mutation is green, D178N mutation is purple.

Site-specific assignments of the protein resonances are requisite for atomic- and residue-level structural studies by NMR spectroscopy. In order to perform residue-specific ^13^C and ^15^N backbone chemical shift assignment of the MoPrP(89-230) fibrils, we recorded four sets of 3D NCOCX, NCACX, CANCO and CONCA spectra on U-^13^C,^15^N-labeled fibrils prepared in U3G1 condition and on U-^13^C,^15^N-labeled, Thr-, Arg-unlabeled samples prepared in all three conditions (Supplementary Figure S4). Analysis of these spectra yielded almost complete assignments for the fibrils formed in U3G1 condition, and partial assignments for the fibrils formed in G2 and G4 conditions. The missing assignments for U3G1 sample include residues Gln186 and Glu200, whereas for G2 and G4 samples, additionally to the selectively unlabeled threonines and arginines, assignments for Glu200, Met205, Glu207 and Asn197, Phe198, Glu200, Glu207, respectively, are missing (Figure 3). Figure 2G–I shows assigned 2D NCA spectra of the fibrils formed in all three conditions. Importantly, the fibrillar cores of the three samples are formed by the same region spanning residues from Pro165 to Ala224, which corresponds to the α-helical C-terminal domain of monomeric PrP including the 3_10_-helical turn of the β2–α2 loop as well as the α2 and α3 helices. This result suggests that the fibrillization propensity of MoPrP(89-230) is a characteristic of the amino acid sequence and does not depend on the particular aggregation condition, implying physiological significance of the identified fibril core region. The identified structured core (residues Pro165–Ala224) of our prepared MoPrP(89-230) fibrils fits very well with the hydrogen-exchange mass spectrometry (HX-MS) data on MoPrP fibrils [50], but does not match with the fibrillar cores found in either of the cryo-EM structures of full-length HuPrP (wt and E196K mutant) or the EPR structure of full-length HuPrP D178N mutant, where they are localized to residues Asn171-Gly230, Asn159-Tyr218 and Phe175-Gln217, respectively [39,40,41]. This could be due to amino acid sequence discrepancies between mouse and human prion proteins, which are mostly localized to the N- and C- terminal regions of the structured core (Figure 3). In agreement with this hypothesis, the fibrillar core of Syrian hamster PrP(23-231) displaying identical sequence near the end and differences in the beginning of fibrillar core region has been assigned by MAS NMR to residues Asn173–Ala224 [38]. In fact, residues between Pro165 and Ser171 are highly variable between different species and previous research has suggested that changes in this part of the sequence induces the species transmissibility barrier [51]. Therefore, these differences in beta strand lengths at the beginning and end of the fibril core may be one of the contributors to the transmissibility barrier.

To determine locations of secondary structure elements, deviations of the assigned ^13^C_α,β_ chemical shifts from random-coil values were calculated (Supplementary Figure S5). The secondary chemical shift analysis shows that all three fibril conformations have similar locations and length of beta strands (Figure 3). Additionally, the chemical shifts of Cys179 and Cys214 indicate disulfide bond formation under all conditions. The structure of fibrils obtained in U3G1 condition comprises eight beta strands formed by residues Val166–His177, Val180–His187, Val189–Lys194, Thr199–Val203, Met206–Glu207, Gln212–Met213, Val215–Gln217 and Lys220–Ala224, two helical turns formed by residues Arg208–Val209 and Tyr218–Gln219. Fibrils obtained in G2 and G4 conditions have identical secondary structure to U3G1 fibrils for the N-terminal residues Val166–His187 and for the C-terminal residues Val215–Ala224 of the fibril core. However, in the central region locations of loop/turn motifs and beta sheets have changed. Here, the G2 fibrils have four beta strands alike the U3G1 fibrils, but comprising residues Val189–Thr192, Asn197–Thr199, Val203–Glu207 and Val209–Met213, whereas the G4 fibrils have two beta strands comprising residues Val189–Lys194, Thr201–Met213. The longer beta strands in G4 fibrils may be correlated with their FTIR signature having the main minimum in the second derivative spectrum at a lower wavenumber indicative of stronger hydrogen bonds. Notably, the helical turn motif involving Tyr218 and Gln219 is present in all samples, indicating that the polypeptide chain makes a turn before the last beta strand. The other helical turn between Arg208 and Val209, on the other hand, is found only in the U3G1 sample, which explains its markedly different Val209 ^13^C_α_ chemical shift in comparison to fibrils prepared in G2 and G4 conditions.

Despite the differences between regions forming the fibril core, the locations of secondary structures partly coincide between the mouse and human prion protein fibrils (Figure 3). The wt HuPrP structure fits best with the MoPrP(89-230) fibril secondary structures, but shows a shorter first beta strand (by 6 residues), C-terminally shifted helical turn (by 2 residues) and elongated last beta strand (by 1 residue) as well as fewer beta strands in the central part between Glu200 and Glu211. Among the three MoPrP(89-230) fibrils, the U3G1 condition fibrils have the most similar secondary structure pattern to wt HuPrP although the latter was prepared in presence of 2 M GdnHCl. Furthermore, prediction of chemical shifts based on the wt HuPrP structure confirms the peculiar Val209 ^13^C_α_ chemical shift observed in the spectra of U3G1 fibrils. The HuPrP E196K mutant shows similarities to the MoPrP(89-230) G2 fibril in the central region. The HuPrP D178N mutant has an additional beta strand at the N-terminus starting at Asn159 and lacks the C-terminal helical turn and beta strand, while in the central part secondary structure motifs fit well to those found in the MoPrP(89-230) G2 and G4 fibrils.

### 2.4. Tertiary Structure Analysis

**Figure 4:**
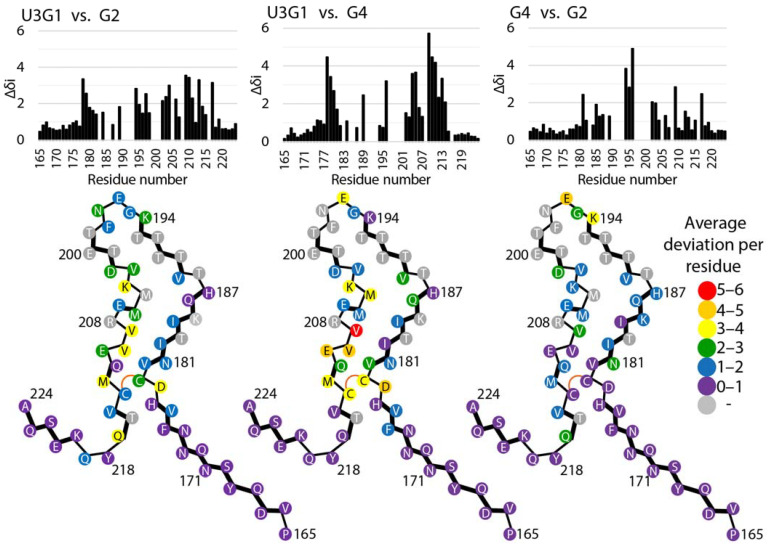
Residue-averaged ^13^C, ^15^N chemical shift differences between MoPrP(89-230) U3G1, G2 and G4 condition fibrils. Top panel: histograms of the chemical shift differences vs. residue number. Bottom panel: structural model of U3G1 fibrils color-coded to reflect the chemical shift differences between U3G1 and G2, U3G1 and G4, G2 and G4 condition fibrils. Beta strands are indicated by bold lines.

Given the high similarity of secondary structures between our MoPrP(89-230) U3G1 and full-length HuPrP fibrils, we created a structural model for the U3G1 fibrils based on the HuPrP cryo-EM structure. Thus, modifications were introduced only in the N- and C-termini, where the first beta strand was extended by 6 residues and the C-terminal helical turn and last beta strand was shifted by 2 residues to match the above-identified MoPrP(89-230) secondary structures. Next, to identify regions exhibiting tertiary structural differences between the MoPrP(89-230) fibrils formed in the three different conditions, we assigned protein side chain resonances based on 2D RFDR and DARR spectra (Supplementary Figure S6) and calculated residue-averaged chemical shift differences of all ^13^C and ^15^N nuclei. Figure 4 shows a graphical representation of the result of this procedure on the structural model of MoPrP(89-230) U3G1 condition fibrils. The differences for residues preceding His177 and succeeding Tyr218 are negligible, indicating that the structure of N- and C-terminal regions of the fibrillar core is not affected by the aggregation condition. Also, as was concluded from the 2D fingerprints, both samples produced with GdnHCl as sole denaturant are more similar to each other and hence show small chemical shift deviations, except for the residues between Lys194 and Glu196, which are part of the protofilament interface of wt HuPrP protein. In contrast, comparisons with the chemical shifts of the U3G1 fibril show larger differences, particularly in the regions Asp178–Asn181 and Lys204–Met213.

**Figure 5:**
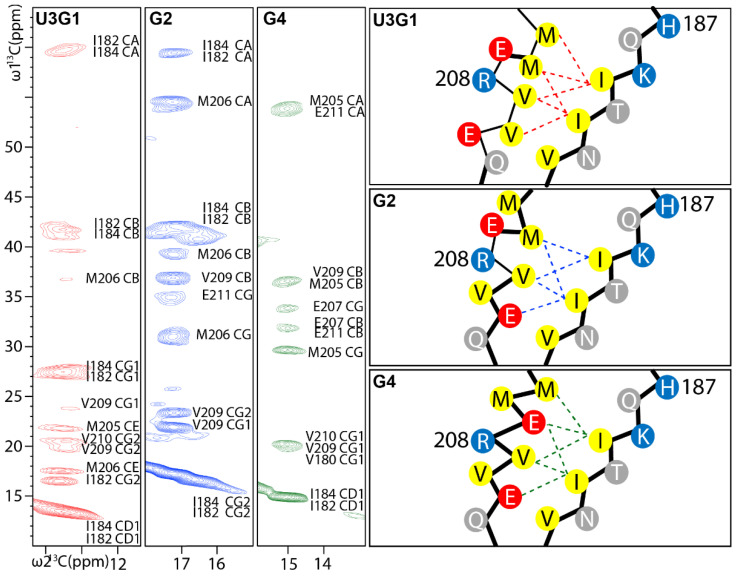
Isoleucine methyl group region of the C(HH)C spectra (mixing time 0.3 ms) of MoPrP(89-230) U3G1 (red), G2 (green) and G4 (blue) condition fibrils. The observed contacts are illustrated on structural models (on the right) created by taking into account the secondary structure information, with beta strands indicated by bold lines.

To gain atomic-level insight into the structural differences of these regions displaying the largest chemical shift variation, we analyzed dipolar recoupling C(HH)C spectra, where several well-resolved cross-peaks in the methyl-group region report on the conformation of Ile182, Ile184, Val209, Val210, Met205, Met206, Glu207 and Glu211 side chains (Figure 5). For U3G1 and G4 fibrils, the most prominent cross-peaks are observed from Cδ1 resonance, while for G2 fibrils, from Cγ2 of Ile182 and/or Ile184. This indicates that the isoleucine side chains are flipped in G2 fibrils in comparison to U3G1 and G4 fibrils. For U3G1 fibrils, the Ile182/184 Cδ1 resonances show contacts with Met205, Met206, Val209 and Val210 side chains indicating that all these residues are facing inwards as seen in the wt HuPrP structure. However, the cross-peak pattern for G2 and G4 fibrils is significantly different. In the spectrum of G2 fibrils, the Cγ2 of Ile182/Ile184 shows contacts with Met206, Val209 and Glu211 side chains implying that the side chains of Met205 and Val210 are facing outwards and the charged Lys204 and Glu211 side chains are facing the core interior. Such an arrangement agrees well with the locations of beta strands and has also been observed in the E196K mutant HuPrP structure [41]. The spectrum of G4 fibrils is again somewhat different and shows contacts from Cδ1 of Ile182/Ile184 to Met205, Glu207, Val209 and Glu211 side chains. This suggests a configuration with both negatively charged residues, Glu207 and Glu211, facing inwards, which also matches the locations of beta strands.

Location of amino acid side chains in amyloid fibril structures is usually determined by the rule of thumb that hydrophobic groups are oriented towards the fibril core, while charged groups are facing the solvent. Our data indicate that the structure of MoPrP(89-230) G2 and G4 fibrils does not follow this rule, since either one (G2) or two (G4) negatively charged glutamic acid side chains are oriented towards the interior of the fibril core. The MoPrP(89-230) assembly into such an unfavorable arrangement seems to be driven by formation of extended beta sheets (i.e., longer beta strands) and avoidance of a helical turn motif. Furthermore, the fact that this destabilizing conformation forms only in presence of high concentration GdnHCl (2 M and 4 M) further suggests that the denaturant is involved in its stabilization. The most obvious difference between our studied aggregation conditions is the ionic strength of the denaturant and, thus, the negative charges in the fibril interior most likely are compensated by ion pair formation with guanidinium. Notably, these tertiary structural differences between the fibrils formed in the three conditions may have further effects on the quaternary structure (i.e., protofilament interactions). Although the G2 and G4 conditions also featured a reduced pH (6.0 compared to 7.4 for U3G1 condition), it most likely did not contribute to the structural differences. The only residue types that could change protonation state in this pH range are histidines (His177 and His187), which are located in the N-terminal region showing identical secondary structures between the three fibrils.

## 3. Materials and Methods

### 3.1. Production of ^13^C, ^15^N Labeled Protein

The expression vector pRSETB harboring nucleic acid sequence encoding N-terminally truncated mouse prion protein (MoPrP(89-230), bold text) fused to an N-terminal linker containing 6×His tail and a thrombin cleavage site (amino acid sequence MRGSHHHHHH GMASLVPRGS **DPGQGGGTHN QWNKPSKPKT NLKHVAGAAA AGAVVGGLGG YMLGSAMSRP MIHFGNDWED RYYRENMYRY PNQVYYRPVD QYSNQNNFVH DCVNITIKQH TVTTTTKGEN FTETDVKMME RVVEQMCVTQ YQKESQAYYD GRRS**), was a generous gift of Prof. Witold K. Surewicz. MoPrP(89-230) expression was done by growing BL21(DE) One Star *E. coli* strain transformed with pRSETB vector in minimal nutrition M9 medium composed of: 6.0 g/L Na_2_HPO_4_, 3.0 g/L KH_2_PO_4_, 1.0 g NaCl, trace metals, 0.10 mM CaCl_2_, 2.0 mM MgSO_4_, 1.0 g/L ^15^N labeled ammonium chloride, 2.0 g/L ^13^C glucose, 1×concentrate Gibco MEM vitamin solution (Life Technologies Europe BV, Bleiswijk, Netherlands), and 0.10 g/L ampicillin. One colony from LB agar plate was inoculated into 20 mL LB medium and grown for 14 h at 37 °C under 180 RPM agitation. Afterwards, 10 mL of this culture was centrifuged at 3000× *g* for 5 min and resuspended into 1 L of M9 medium. The culture was grown for approximately 8 h at 37 °C under 220 RPM agitation until the optical density at 600 nm reached 0.6–0.9. The expression was induced with 0.50 mM of IPTG, and the culture was grown for an additional 16 h at 20 °C under 220 RPM agitation.

The selectively unlabeled protein (natural abundance Thr and Arg, ^13^C, ^15^N isotopes in the other amino acid residues) was expressed as described above, but the culture harvested from 10 mL LB medium was suspended and grown in M9 medium with 0.20 g/L of each Arg and Thr amino acids. The selective unlabeling efficiency was determined by solution NMR to be above 90% by comparing peak intensities in ^1^H-^15^N HSQC spectra of Thr-, Arg-unlabeled and fully labeled samples (Supplementary Figures S1 and S2). Expressed MoPrP(89-230) was purified as described previously [31,43]. The only difference from the described protocol was, that 6×His-tag was not cleaved from the protein.

### 3.2. Aggregation of Labeled MoPrP(89-230) into Fibrils

The prion protein fibrils were prepared in three different conditions. For two conditions, lyophilized MoPrP(89-230) was dissolved in 50 mM sodium phosphate buffer (pH 6.0), containing either 2 M or 4 M GdnHCl, while the third condition the sample was dissolved in PBS buffer (pH 7.4) containing 3 M Urea and 1 M GdnHCl. In all cases the final protein concentration was 0.5 mg/mL (~27 µM). The solutions were then distributed into 1.5 mL test-tubes (1.0 mL final volume) and incubated at 37 °C and 220 RPM agitation (test-tubes were placed horizontally on the shaken surface) for three (2 M GdnHCl and 1 M GdnHCl with 3 M Urea samples) or seven (4 M GdnHCl samples) days. The formed aggregates were sonicated for 10 min on ice using a Sonopuls 3100 ultrasonic homogenizer (Bandelin, Berlin, Germany; MS-72 tip, 20% of total power, using 30 s rest and 30 s sonication cycles). 1 mL of each sonicated aggregate solution was mixed with 1 mL of fresh, non-aggregated protein solutions (identical to their initial solutions) and incubated at 60 °C under quiescent conditions for 2 h. The aggregate sonication and reseeding steps were repeated 5 times, each time mixing the aggregate solution with fresh protein solution in equal volumes, resulting in a final volume of 32 mL. Subsequently, the aggregate solution was distributed into 16 × 1.5 mL test-tubes and centrifuged at 20,000× *g*, 20 °C for 30 min. The supernatant was removed, and all fibrils were combined into one 1.5 mL test-tube and resuspended in 1 mL of their respective initial buffer for further analysis. The obtained fibrils were packed in 3.2 mm MAS NMR rotors by ultra-centrifugation at 4 °C and 110,000× *g* overnight using a rotor packing device from Giotto Biotech (Sesto Fiorentino, Italy).

### 3.3. Fourier-Transform Infrared (FTIR) Analysis of Aggregates

FTIR measurements were performed as described previously [31,52]. In brief, fibril samples were centrifuged at 20,000× *g*, 20 °C for 30 min and resuspended into 1 mL of D_2_O. This centrifugation and resuspension procedure was repeated 4 times, with the final suspension volume of 0.20 mL. The samples were then sonicated for 20 s using a MS-72 tip (20% of total power, constant sonication). FTIR spectra were recorded using 256 interferograms per spectrum with 2 cm^−1^ resolution and analyzed using GRAMS software (Thermo Fisher Scientific, Waltham, MA, USA).

### 3.4. AFM Imaging of Aggregates

AFM measurements were performed as described previously [53]. Briefly, 20 µL of each sample were deposited on freshly cleaved mica and incubated for 1 min. Subsequently, the samples were washed with 2 mL of Milli-Q water and dried under gentle airflow. Three-dimensional sample maps were acquired with Bruker Dimension Icon (Bruker, Billerica, MA, USA) atomic force microscope operating in tapping mode and equipped with a silicon cantilever RTESPA-300 (Bruker, Billerica, MA, USA). All images were acquired at high-resolution (1024 × 1024 pixels). Three-dimensional AFM maps were flattened using SPIP software (Image Metrology A/S, Lyngby, Denmark). The height of the fibrils was determined from line profiles taken perpendicular to the fibril axes [54].

### 3.5. MAS NMR Spectroscopy

All spectra were recorded on an 800 MHz Bruker Avance III HD spectrometer (Bruker, Billerica, MA, USA) equipped with a 3.2 mm H/C/N E-free MAS probe. For all the samples in this project 1D ^13^C cross-polarization (CP), ^15^N CP, ^13^C INEPT, 2D NCA, NCO, DARR, RFDR and C(HH)C, 3D NCACX, NCOCX, CANCO, and CONCA spectra were acquired. The MAS frequency was 17 kHz during acquisition of 2D DARR, RFDR and C(HH)C spectra, while for other spectra it was 12.5 kHz. Temperature regulation was set to 273 K. Spectra acquisition and processing parameters (pulse lengths, amplitudes, delays, acquisition time, number of scans and window function) are summarized in Supplementary Tables S1–S4. Spectra were processed with Bruker Topspin 3.57 and analyzed in CARA [55]. Secondary structure analysis was performed by calculating deviations of ^13^C_α,β_ chemical shifts from random coil values [56] using the equation Δδ_i_ = (ΔδCα_i_ − ΔδCβ_i_).

## 4. Conclusions

We have prepared MoPrP(89-230) fibrils in three different conditions widely used for studies of prion proteins and have structurally characterized them using AFM, FTIR and MAS NMR spectroscopy. The backbone and side chain resonances have been assigned for most residues through analysis of 2D and 3D MAS NMR spectra and locations of secondary structures have been identified though secondary chemical shift analysis. The results show that regardless of the aggregation condition the MoPrP(89-230) fibrillar core is comprised by residues Pro165 to Ala224, which highlights the physiological significance of this protein region and agrees well with earlier results obtained by HX-MS. However, it does not match with the fibrillar core of human or Syrian hamster prion proteins, which could be due to differences in their amino acid sequence. Analysis of chemical shift differences between the MoPrP(89-230) fibrils obtained in the three conditions points to a nearly identical structure for the N- and C-terminal parts of the fibril core, while the largest structural differences are found in the central region near residues Asp178–Asn181 and Lys204–Met213. Further analysis of dipolar recoupling ssNMR spectra indicates that the U3G1 fibrils have a structure that closely resembles the wt HuPrP fibrils prepared in presence of 2 M GdnHCl. The spectra for G2 and G4 fibrils reveal an altered structure with one or two glutamic acid residues facing inwards, which results in formation of longer beta strands and absence of a helical turn motif. Altogether, these findings suggest that the role of the denaturant in driving MoPrP(89-230) aggregation into a particular fibrillar structure is related to its ability to stabilize fibril core interior-facing glutamic acid residues though charge screening. Therefore, the fibrils obtained in presence of high concentrations of the charged GdnHCl denaturant may be less biologically relevant due to significant deviation from physiological ionic strength. The aggregation conditions could have further effects on the quaternary structure as the protofilament interactions often involve inter-chain salt bridges. To study these effects, preparation of new fibril samples with mixed ^13^C/^15^N labels is required, which is beyond the scope of this study.

## Data Availability

Assigned chemical shifts of U-^13^C,^15^N-labeled, Thr-, Arg-unlabeled prion protein fibrils obtained in 2 M GdnHCl with 50 mM sodium phosphate (pH 6.0), in 4 M GdnHCl with 50 mM sodium phosphate (pH 6.0), and in 3 M urea, 1 M GdnHCl, 1× PBS (pH 7.4) have been deposited in the Biological Magnetic Resonance Data Bank under accession codes 50950, 50951 and 50952, respectively. All other data can be provided upon request.

## Supplementary Materials

The following are available online at https://www.mdpi.com/article/10.3390/ijms22179635/s1.
