# Supplementary material for: Aggregation Condition–Structure Relationship of Mouse Prion Protein Fibrils
Source: Int J Mol Sci. 2021 Sep 6;22(17):9635. doi: 10.3390/ijms22179635 (PMC8431800; doi:10.3390/ijms22179635)
Supplement: Supplementary file 1 [file ijms-22-09635-s001.zip › ijms-1366001-supplementary.pdf]

International Journal of *Molecular Sciences*

MDPI

Supplementary Materials

Aggregation Condition–Structure Relationship of Mouse Prion Protein Fibrils

Jēkabs Fridmanis [1], Zigmantas Toleikis [1,2], Tomas Sneideris [2,†], Mantas Ziaunys [2], Raitis Bobrovs [1], Vytautas Smirnovas [2] and Kristaps Jaudzems [1,*]

1 Department of Physical Organic Chemistry, Latvian Institute of Organic Synthesis, Aizkraukles 21, LV-1006 Riga, Latvia; fridmanis.jekabs@osi.lv (J.F.); zigmantas.toleikis@gmc.vu.lt (Z.T.); raitis.bobrovs@osi.lv (R.B.)
2 Institute of Biotechnology, Life Sciences Center, Vilnius University, LT-10257 Vilnius, Lithuania; sneideris.t@gmail.com (T.S.); mantas.ziaunys@gmc.vu.lt (M.Z.); vytautas.smirnovas@bti.vu.lt (V.S.)
* Correspondence: kristaps.jaudzems@osi.lv
† Present Address: Yusuf Hamied Department of Chemistry, University of Cambridge, Cambridge CB2 1EW, UK.

Table of Contents

Figure S1........ 2
Figure S2........ 3
Figure S3........ 4
Figure S4........ 5
Figure S5........ 6
Figure S6........ 7
Table S1........ 8
Table S2........ 9
Table S3........ 10
Table S4........ 11

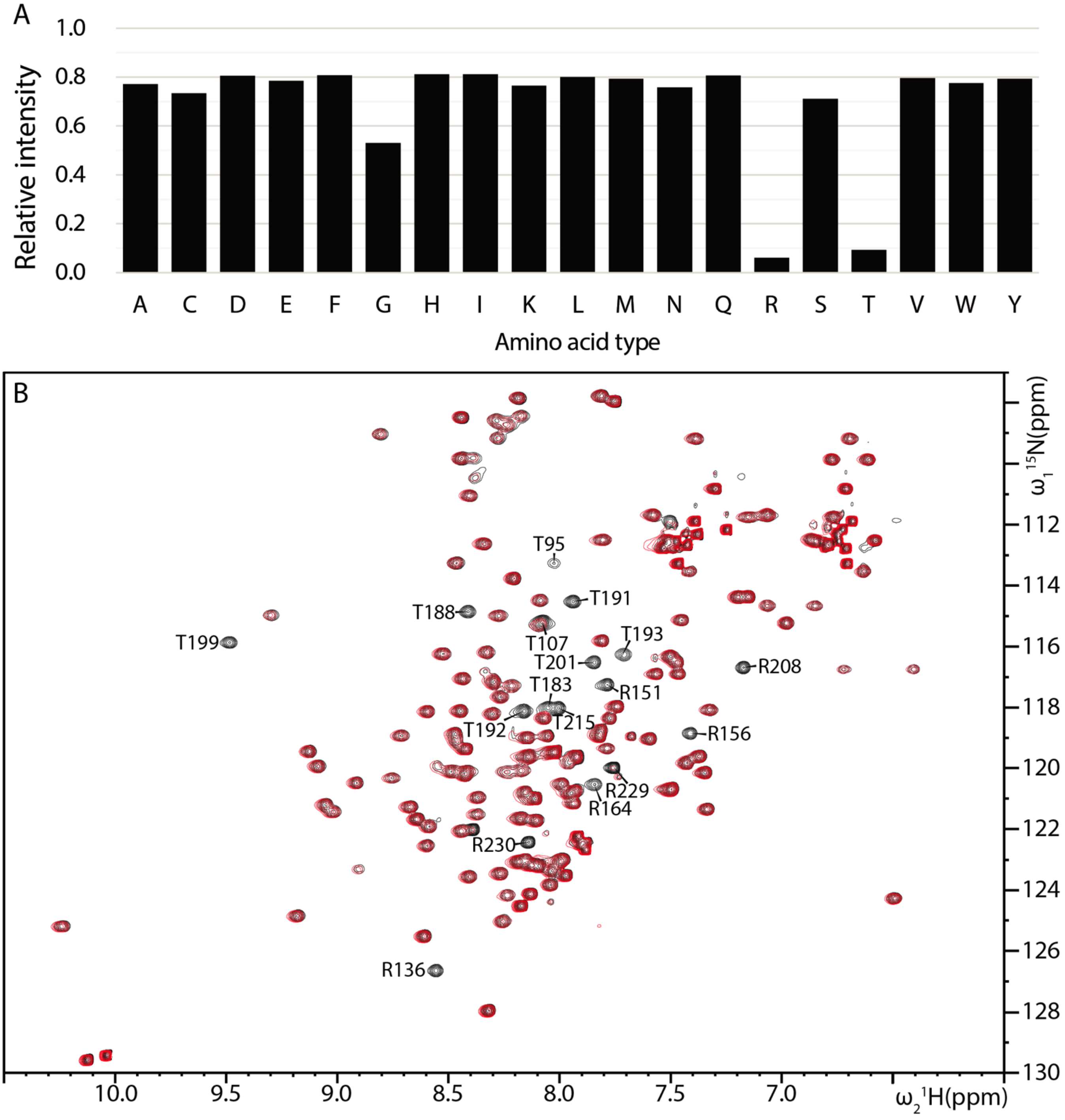

Figure S1. Amino acid-selective unlabeling of MoPrP(89-230) analyzed by solution NMR. (**A**) Average peak intensities of each amino acid residue type in ^{15}N-^{1}H HSQC spectra of MoPrP(89-230) samples selectively unlabeled by adding 0.2 g of natural abundance Arg and Thr amino acids per liter of M9 medium relative to uniformly ^{13}C, ^{15}N-labeled samples. (**B**) Overlay of ^{15}N-^{1}H HSQC spectra of fully ^{13}C, ^{15}N-labeled (black) and Thr-, Arg-selectively unlabeled (red) MoPrP(90-230) samples.

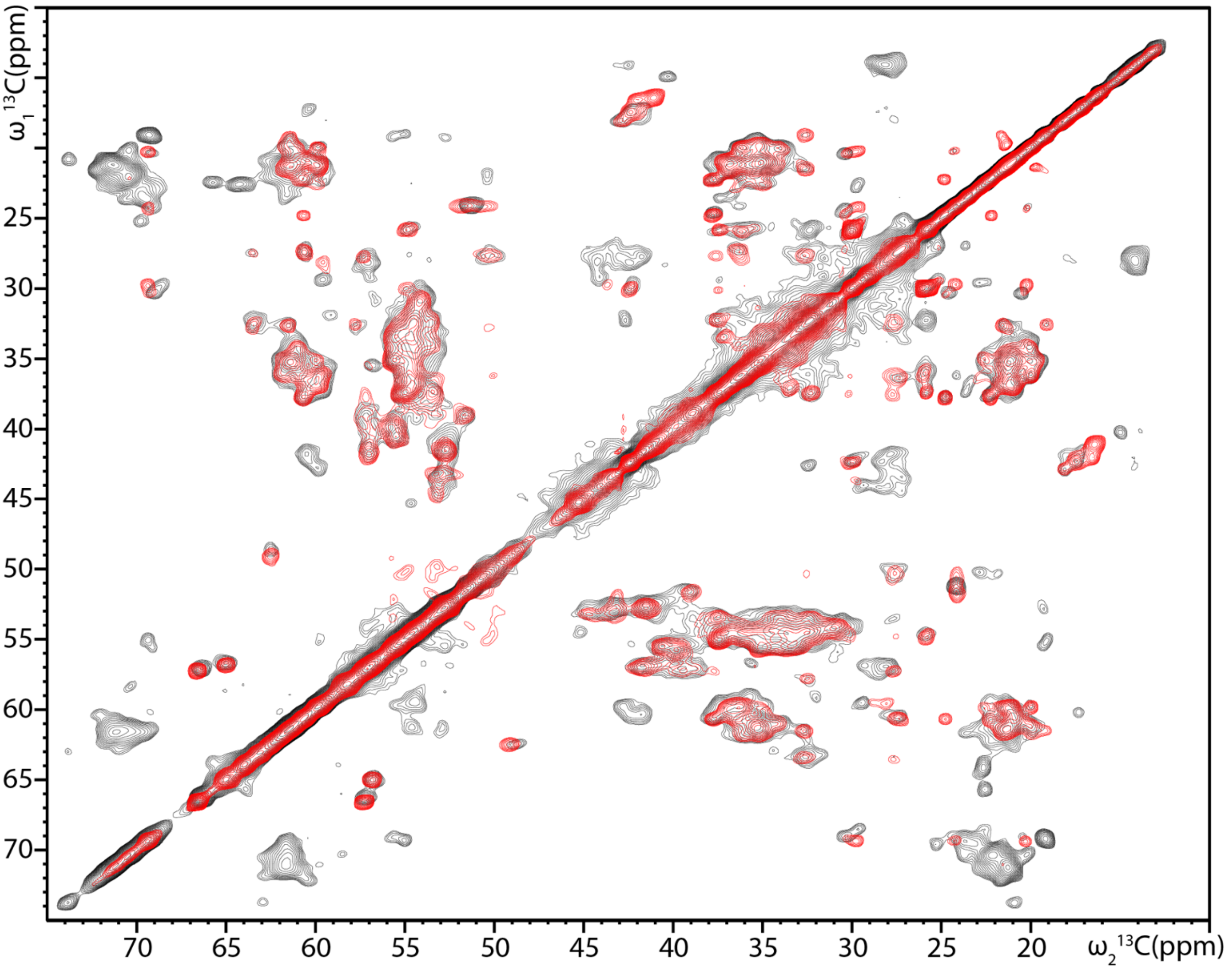

Figure S2. Amino acid-selective unlabeling of MoPrP(89-230) analyzed by solid-state NMR. Overlay of 2D DARR MAS NMR spectra of U3G1 fibrils prepared from fully ^{13}C, ^{15}N-labeled (black) and Thr-, Arg-selectively unlabeled (red) MoPrP(90-230) samples. The spectra were recorded at 800 MHz at 273 K with 17 kHz MAS.

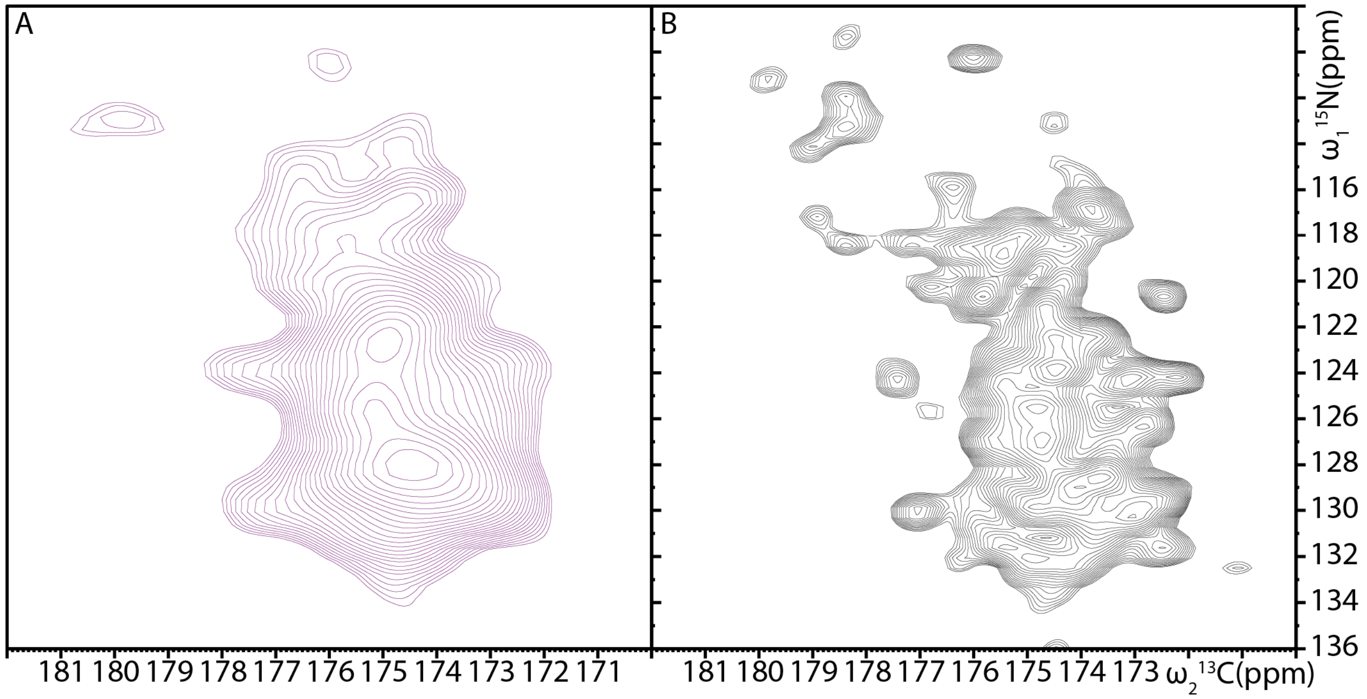

Figure S3. Comparison of spontaneously aggregated and sixth-generation reseeded fully ^{13}C, ^{15}N-labeled MoPrP(89-230) U3G1 condition fibrils. (**A**) 2D NCO spectrum of spontaneously aggregated and (**B**) 2D NCO spectrum of sixth-generation fibrils.

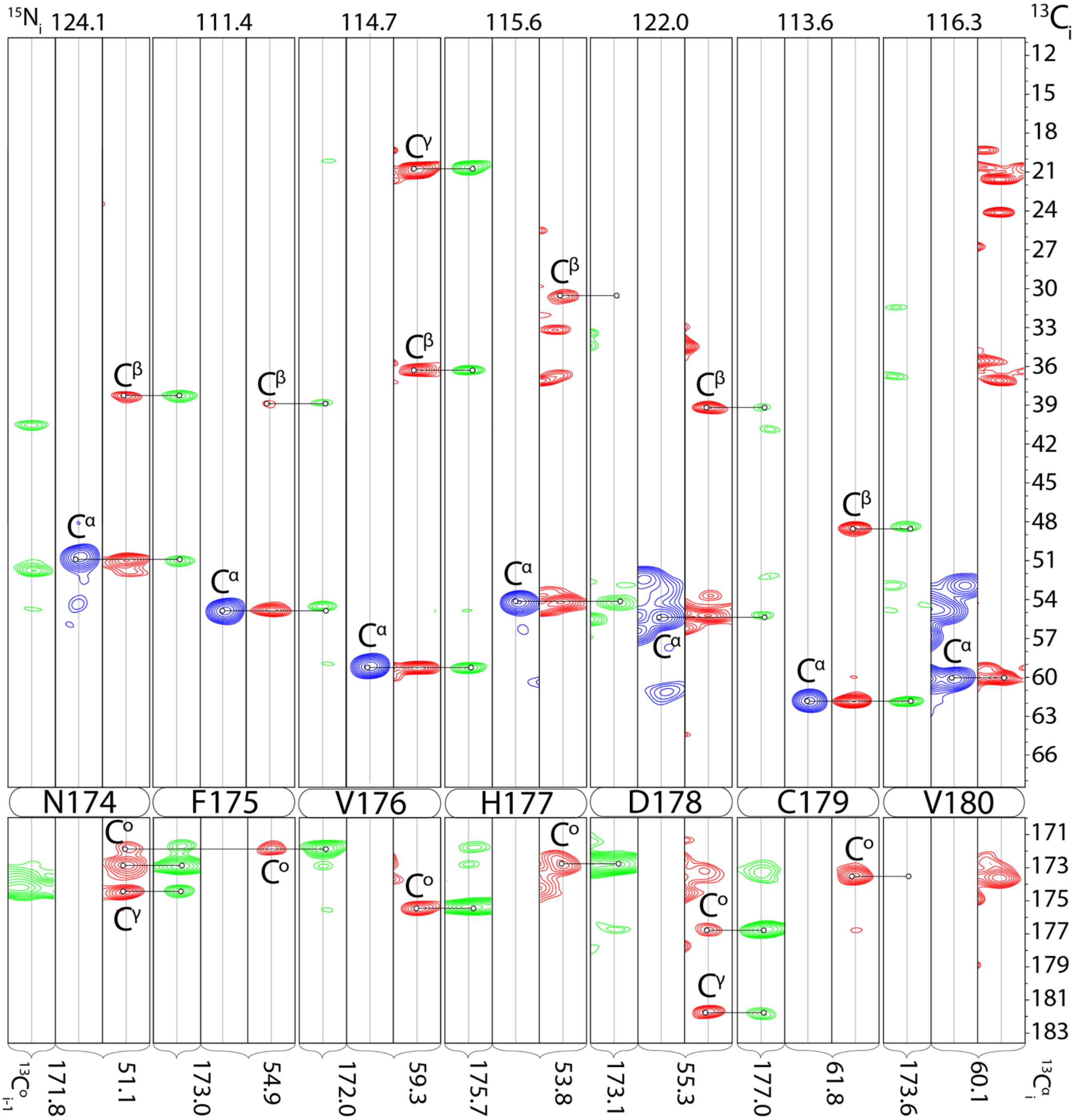

Figure S4. Illustration of backbone assignment of MoPrP(89-230) G2 fibrils. Representative strip plot of NCACX (red), CANCO (blue), and NCOCX (green) spectra recorded at 800 MHz and 273 K with 12.5 kHz MAS, mixing time 65 ms (DARR).

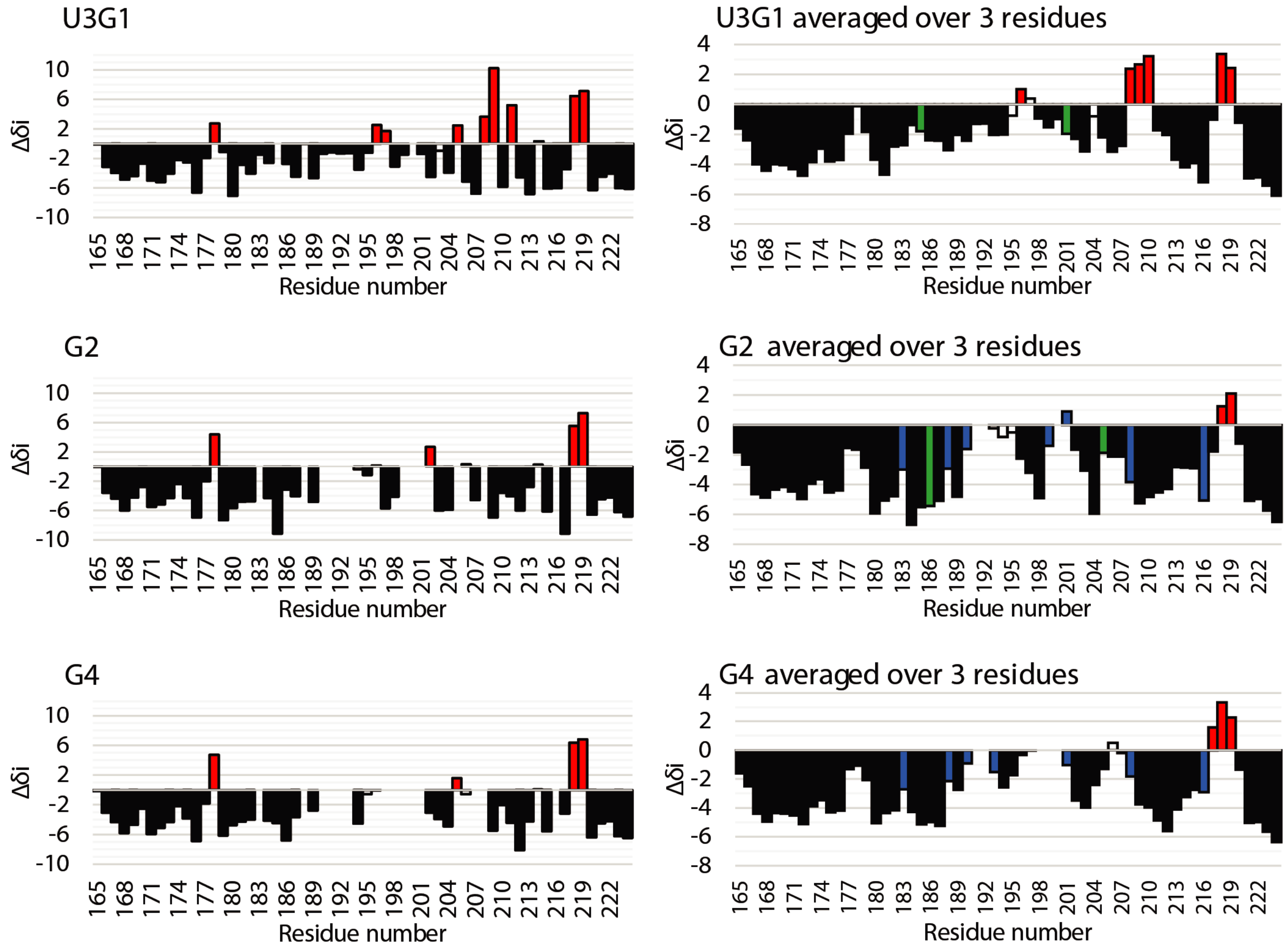

Figure S5. Secondary chemical shift analysis of MoPrP(89-230) U3G1, G2 and G4 fibrils. Here $\Delta\delta_i$ is calculated as ($\Delta\delta C\alpha_i - \Delta\delta C\beta_i$) on the left and as ($\Delta\delta C\alpha_i - \Delta\delta C\beta_i$) + ($\Delta\delta C\alpha_{i-1} - \Delta\delta C\beta_{i-1}$) + ($\Delta\delta C\alpha_{i+1} - \Delta\delta C\beta_{i+1}$) on the right, where results are averaged over 3 residues. The one-residue values were used for determination of secondary structures for the assigned residues, whereas values calculated over three residues were used to predict the secondary structures for isolated unassigned residues. $\Delta\delta_i$ values above 1 are indicative of alpha helix (red), and below -1 of beta sheet (black), whereas $\Delta\delta_i$ values between -1 and 1 indicate non-regular structure (white). Selectively unlabeled residues are indicated with blue bars and residues with missing assignment are indicated with green bars.

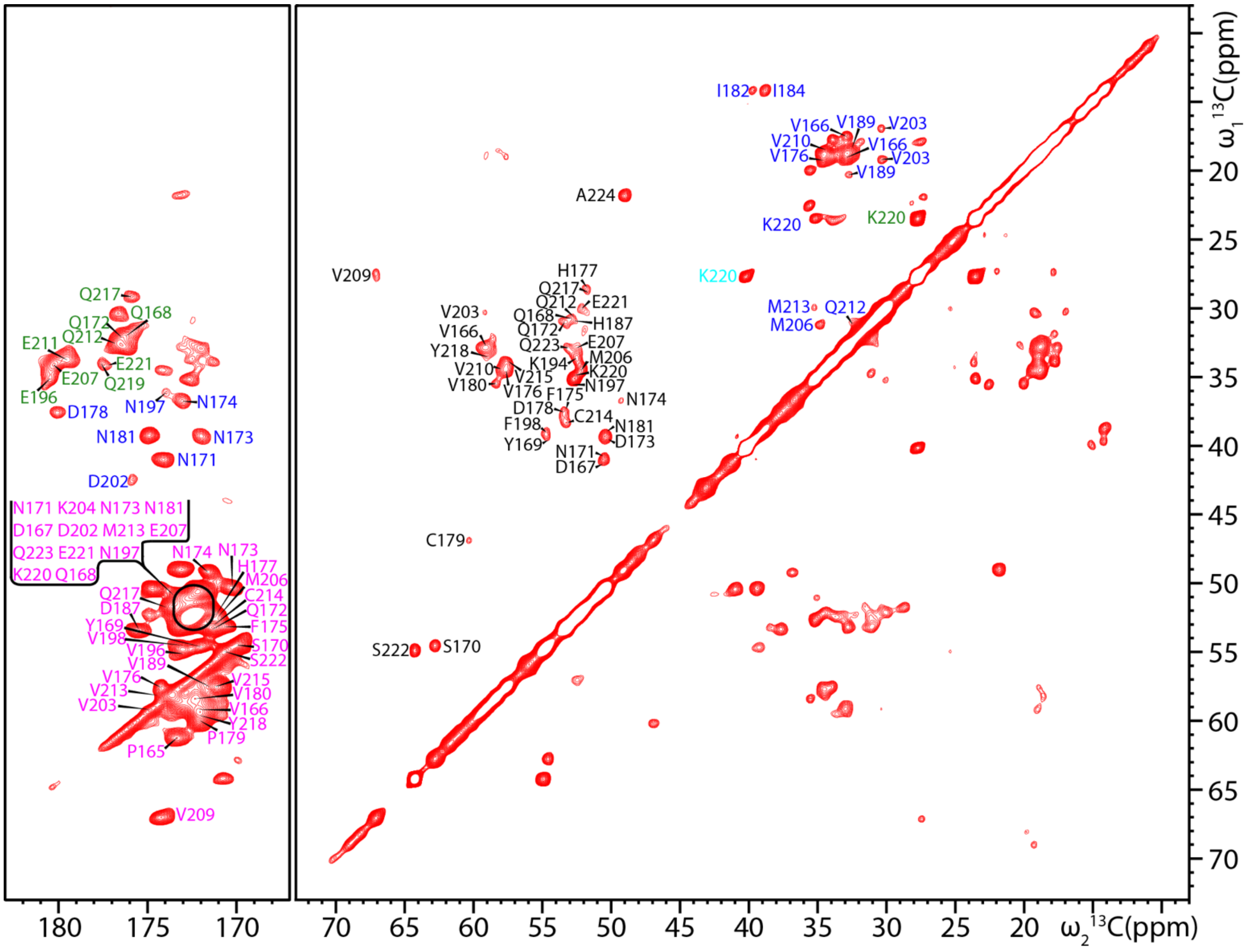

Figure S6. Assigned 2D RFDR MAS NMR spectrum of Thr-, Arg-selectively unlabeled MoPrP(89-230) U3G1 fibrils. The spectra were recorded at 800 MHz and 273 K with 17 kHz MAS.

Table S1. Acquisition and processing parameters for the MAS NMR spectra of fully-labeled MoPrP(89-230) U3G1 fibrils.

Spectrum	MAS Freq. (kHz)	Dec. Field Ampl. (kHz)	Max Indirect Evolution (ms)	Rec. Delay (s)	Scans	Exp. Time	Window Function
2D ^{13}C-^{13}C DARR (15 ms mixing)	17	83.3	9.9	2.5	32	18 h 15 min	60° shifted sine-bell squared
2D ^{13}C-^{13}C RFDR (1.6 ms mixing)	17	83.3	7.7	2.5	32	13 h 56 min	60° shifted sine-bell squared
2D C(HH)C (0.3 ms mixing)	17	83.3	5.5	2.3	64	33 h 26 min	60° shifted sine-bell squared
2D NCO	12.5	72.0	14.1	2	64	2 h 18 min	60° shifted sine-bell squared
2D NCA	12.5	72.0	10.6	2	64	1 h 43 min	60° shifted sine-bell
3D CONCA	12.5	72.0	5.9 (CO)<br>5.3 (N)	2.5	64	28 h 4 min	90° shifted sine-bell
3D NCOCX (65 ms mixing)	12.5	72.0	9.2 (N)<br>10.8 (CO)	2	192	257 h 33 min	90° shifted sine-bell squared
3D NCACX (65 ms mixing)	12.5	72.0	7.9 (N)<br>3.9 (CA)	2.5	192	228 h 58 min	90° shifted sine-bell
3D CANCO	12.5	72.0	3.9 (CA)<br>7.9 (N)	2.5	64	74 h 32 min	90° shifted sine-bell

Table S2. Acquisition and processing parameters for the MAS NMR spectra of Thr-, Arg-selectively-unlabeled MoPrP(89-230) U3G1 fibrils.

Spectrum	MAS Freq. (kHz)	Dec. Field Ampl. (kHz)	Max Indirect Evolution (ms)	Rec. Delay (s)	Scans	Exp. Time	Window Function
2D ^{13}C-^{13}C DARR (15 ms mixing)	17	83.3	7.7	2.5	32	14 h 8 min	60° shifted sine-bell squared
2D ^{13}C-^{13}C RFDR (1.6 ms mixing)	17	83.3	7.7	2.5	32	13 h 56 min	60° shifted sine-bell squared
2D C(HH)C (0.3 ms mixing)	17	83.3	7.7	2.5	256	112 h 26 min	60° shifted sine-bell squared
2D NCO	12.5	72.0	14.1	2	128	4 h 37 min	60° shifted sine-bell squared
2D NCA	12.5	72.0	14.1	2	128	4 h 37 min	60° shifted sine-bell
3D CONCA	12.5	72.0	5.9 (CO)<br>5.3 (N)	2.5	320	140 h 20 min	90° shifted sine-bell
3D NCOCX (65 ms mixing)	12.5	72.0	10.6 (N)<br>10.8 (CO)	2	256	343 h 24 min	90° shifted sine-bell squared
3D NCACX (65 ms mixing)	12.5	72.0	7.9 (N)<br>3.9 (CA)	2.5	192	228 h 57 min	90° shifted sine-bell
3D CANCO	12.5	72.0	3.9 (CA)<br>7.9 (N)	2.5	192	223 h 30 min	90° shifted sine-bell

Table S3. Acquisition and processing parameters for the MAS NMR spectra of Thr-, Arg-selectively-unlabeled MoPrP(89-230) G2 fibrils.

Spectrum	MAS Freq. (kHz)	Dec. Field Ampl. (kHz)	Max Indirect Evolution (ms)	Rec. Delay (s)	Scans	Exp. Time	Window Function
2D ^{13}C-^{13}C DARR (15 ms mixing)	17	83.3	7.7	2.5	32	14 h 8 min	60° shifted sine-bell squared
2D ^{13}C-^{13}C RFDR (1.6 ms mixing)	17	83.3	7.7	2.5	32	13 h 56 min	60° shifted sine-bell squared
2D C(HH)C (0.3 ms mixing)	17	83.3	7.7	2.5	384	168 h 40 min	60° shifted sine-bell squared
2D NCO	12.5	72.0	14.1	2	128	4 h 37 min	60° shifted sine-bell squared
2D NCA	12.5	72.0	14.1	2	64	2 h 18 min	60° shifted sine-bell
3D CONCA	12.5	72.0	5.9 (CO)<br>5.3 (N)	2.5	64	28 h 4 min	90° shifted sine-bell
3D NCOCX (65 ms mixing)	12.5	72.0	10.6 (N)<br>10.8 (CO)	2	64	85 h 54 min	90° shifted sine-bell squared
3D NCACX (65 ms mixing)	12.5	72.0	7.9 (N)<br>3.9 (CA)	2.5	64	76 h 18 min	90° shifted sine-bell
3D CANCO	12.5	72.0	3.9 (CA)<br>7.9 (N)	2.5	64	74 h 32 min	90° shifted sine-bell

Table S4. Acquisition and processing parameters for the MAS NMR spectra of Thr-, Arg-selectively-unlabeled MoPrP(89-230) G4 fibrils.

**Spectrum**	**MAS Freq. (kHz)**	**Dec. Field Ampl. (kHz)**	**Max Indirect Evolution (ms)**	**Rec. Delay (s)**	**Scans**	**Exp. Time**	**Window Function**
2D ^{13}C-^{13}C DARR (15 ms mixing)	17	83.3	7.7	2.5	64	28 h 17 min	60° shifted sine-bell squared
2D ^{13}C-^{13}C RFDR (1.6 ms mixing)	17	83.3	7.7	2.5	32	13 h 56 min	60° shifted sine-bell squared
2D C(HH)C (0.3 ms mixing)	17	83.3	7.7	2.5	384	168 h 40 min	60° shifted sine-bell squared
2D NCO	12.5	72.0	14.1	2	64	2 h 18 min	60° shifted sine-bell squared
2D NCA	12.5	72.0	14.1	2	64	2 h 18 min	60° shifted sine-bell
3D CONCA	12.5	72.0	5.9 (CO)<br>5.3 (N)	2.5	128	56 h 7 min	90° shifted sine-bell
3D NCOCX (65 ms mixing)	12.5	72.0	10.6 (N)<br>10.8 (CO)	2	256	343 h 20 min	90° shifted sine-bell squared
3D NCACX (65 ms mixing)	12.5	72.0	7.9 (N)<br>3.9 (CA)	2.5	256	305 h 10 min	90° shifted sine-bell
3D CANCO	12.5	72.0	3.9 (CA)<br>7.9 (N)	2.5	128	149 h 52 min	90° shifted sine-bell
